# A Hierarchical-Based Learning Approach for Multi-Action Intent Recognition

**DOI:** 10.3390/s24237857

**Published:** 2024-12-09

**Authors:** David Hollinger, Ryan S. Pollard, Mark C. Schall, Howard Chen, Michael Zabala

**Affiliations:** 1 Department of Mechanical Engineering, Auburn University, Auburn, AL 36849, USA; dzh0063@auburn.edu (D.H.); rzp0051@auburn.edu (R.S.P.); zabalme@auburn.edu (M.Z.); 2 Department of Industrial & Systems Engineering, Auburn University, Auburn, AL 36849, USA; mcs0084@auburn.edu; 3 Department of Industrial & Systems Engineering and Engineering Management, University of Alabama-Huntsville, Huntsville, AL 35899, USA

**Keywords:** wearable sensors, accelerometers, gyroscopes, movement intent prediction

## Abstract

Recent applications of wearable inertial measurement units (IMUs) for predicting human movement have often entailed estimating action-level (e.g., walking, running, jumping) and joint-level (e.g., ankle plantarflexion angle) motion. Although action-level or joint-level information is frequently the focus of movement intent prediction, contextual information is necessary for a more thorough approach to intent recognition. Therefore, a combination of action-level and joint-level information may offer a more comprehensive approach to predicting movement intent. In this study, we devised a novel hierarchical-based method combining action-level classification and subsequent joint-level regression to predict joint angles 100 ms into the future. K-nearest neighbors (KNN), bidirectional long short-term memory (BiLSTM), and temporal convolutional network (TCN) models were employed for action-level classification, and a random forest model trained on action-specific IMU data was used for joint-level prediction. A joint-level action-generic model trained on multiple actions (e.g., backward walking, kneeling down, kneeling up, running, and walking) was also used for predicting the joint angle. Compared with a hierarchical-based approach, the action-generic model had lower prediction error for backward walking, kneeling down, and kneeling up. Although the TCN and BiLSTM classifiers achieved classification accuracies of 89.87% and 89.30%, respectively, they did not surpass the performance of the action-generic random forest model when used in combination with an action-specific random forest model. This may have been because the action-generic approach was trained on more data from multiple actions. This study demonstrates the advantage of leveraging large, disparate data sources over a hierarchical-based approach for joint-level prediction. Moreover, it demonstrates the efficacy of an IMU-driven, task-agnostic model in predicting future joint angles across multiple actions.

## 1. Introduction

Human movement encompasses a variety of dynamic actions, such as walking, running, jumping, and pivoting [1]. Recognition of human intent is useful for controlling wearable robots [2], detecting abnormal movement patterns [3], and providing feedback for gait retraining [4]. Researchers have used pattern recognition algorithms to capture the versatility of human movement and adapt assistive devices to amplify performance [5]. Furthermore, intent recognition algorithms that generalize across multiple daily activities can significantly enhance user–device integration [6,7]. Factors influencing movement intent include the type of action (e.g., walking, jogging, stair ascent, stair descent, kneeling) [8], environmental conditions (e.g., slopes, terrain) [9], and physiological characteristics (e.g., musculoskeletal geometry) [10], as well as the type, number, and placement of sensors [11,12]. Controllers of wearable devices often incorporate a combination of these user-dependent and user-independent factors to assist the user [2,9,13,14]. Specifically, kinematic and kinetic inputs fed into pattern recognition algorithms can provide user-specific control of a wearable exoskeleton [15,16]. Given the versatility of dynamic human movements, previous studies have focused on developing robotic controllers to predict movement intent across a wide range of actions [8,9,17]. For example, soldiers encounter a wide range of activities during operational tasks, and predicting their motion across these tasks may be beneficial for effective exoskeleton control. Additionally, rehabilitation devices must quickly adjust to the user’s intended motion from one action to the next [18]. A wearable robotic controller based on movement intent would ideally precede human motion by approximately 100 ms, which is the time between detection of muscle activation and limb motion [19,20], thereby providing naturalistic assistance to the user [6,7,21]. 

Machine learning offers a flexible solution for predicting movement intent, by mapping a sequence of input signals (e.g., accelerometry and angular velocity) to a sequence of target signals (e.g., joint angles). Models can be trained to recognize human activities at the action level (e.g., walking, running, kneeling) and predict movement intent at the joint level (e.g., ankle plantarflexion angle). One prominent approach for handling sequential data in movement prediction is the use of convolutional architectures, such as a temporal convolutional network (TCN). TCNs have shown success in handling sequential data for trajectory prediction tasks [22] and human motion prediction [23]. Additionally, bidirectional long short-term memory (BiLSTM) networks have shown promise in predicting future joint angles during level-ground walking [24]. Although these complex deep learning models offer high prediction accuracy, they are also computationally expensive, potentially consuming significant storage space and causing runtime delays. Therefore, it is worth comparing their performance to simpler models, such as k-nearest neighbors and random forest. A comparison of machine learning models based on varied complexities could help determine the optimal approach for using wearable IMU sensors to predict joint angles.

Depending on the application, some machine learning models are trained to recognize a wide range of activities [2,8] while others are optimized for a single activity, such as walking [25]. A single joint-level model trained on many actions, which can be referred to as an “action-generic model”, directly predicts joint angles, no matter the action. These models are robust enough to predict joint angles across a broad range of activities, but may not be sufficiently accurate for some applications [2,13].

“Action-specific models” are machine learning models trained only with data of that specific action and can achieve high accuracy when predicting joint angles for that specific action [24]. However, action-specific models may struggle to generalize with regard to actions outside their training set. This is due to the bias–variance tradeoff in machine learning, where the most accurate algorithms are not always the most reliable [26,27]. 

The tradeoff between achieving modest prediction accuracy across a wide range of actions and high accuracy for a limited number of actions presents a challenge for applications that aim to predict human intent. To overcome this limitation, an action classifier that identifies the user’s action *a priori* may provide relevant information about their current state, potentially improving the accuracy of subsequent joint-angle predictions. This combination of action-level and joint-level models for multi-tiered motion prediction can be referred to as a “hierarchal-based approach”. This approach utilizes an action classifier to first identify the user’s activity, which then triggers the correct action-specific joint-angle prediction model to be used. In 2024, Lim et al. employed a hierarchical-based approach to estimate relative hand load by first classifying workstation height (floor vs. knee) followed by classifying the handling mode. After these classifications, they used a generalized linear model to estimate relative hand load for each specific combination of workstation height and handling mode [28]. Their study found that the hierarchical-based approach provided greater accuracy in estimating hand load compared with the general linear model (mean absolute errors of 5.6–5.8% vs. 8.5–8.7%) [28]. Although a hierarchical-based approach can indicate which action-specific model should be used, such methods run the risk of misclassifying actions, compounding prediction error at the joint level. To our knowledge, no study has yet explored the feasibility of a hierarchical-based approach that combines action-level and joint-level models for predicting future joint angles across multiple actions. 

Therefore, this study aimed to develop an optimal wearable-IMU-driven method of detecting movement intent by comparing action-generic, action-specific, and hierarchical-based approaches for accurate and reliable prediction of joint angles across multiple actions. This study focused on five distinct actions (e.g., backward walking, kneeling down, kneeling up, running, and walking) with clear movement patterns, to minimize confusion during transitional states, regardless of speed. We hypothesized that the action-specific model would have greater joint angle prediction accuracy than the action-generic model. Additionally, since action-level models provide relevant information for subsequent joint-level models, we hypothesized that combining these models (i.e., a hierarchical-based approach) would result in greater joint angle prediction accuracy than using an action-generic joint-level prediction model alone. Therefore, the goal of this study was to determine which machine learning approach (e.g., action-generic, action-specific, or hierarchical-based) could provide the greatest accuracy for predicting future joint angles across multiple activities. The major contributions of this work are as follows:

1. The development of a novel hierarchical-based method that integrates action-level classification with joint-level regression to predict future joint angles from IMU inputs;
2. A systematic comparison of action-generic, action-specific, and hierarchical-based models for predicting joint angles;
3. The evaluation of model performance across five distinct actions (e.g., backward walking, kneeling down, kneeling up, running, and walking) to assess generalizability and accuracy.

## 2. Materials and Methods

**Figure 1:**
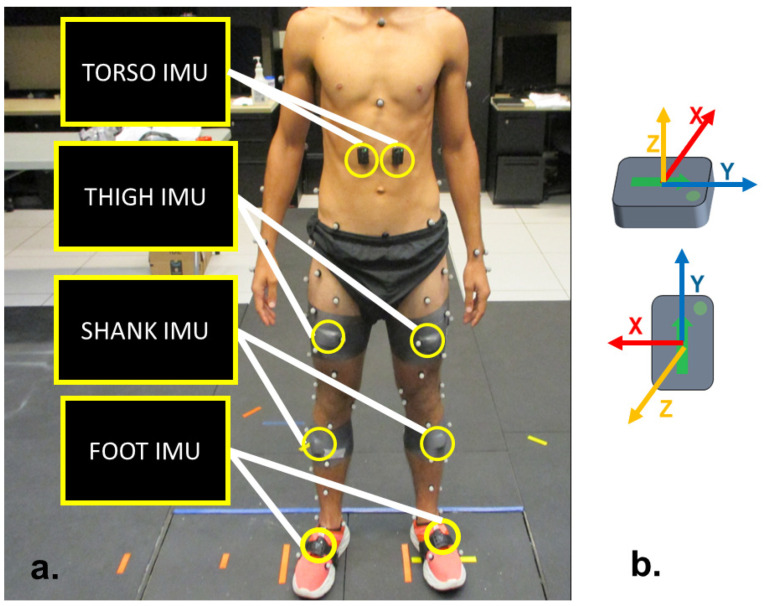
(**a**) Experimental instrumentation setup. Motion-capture marker trajectories were converted to joint angles using inverse kinematics in Visual3D. (**b**) Eight IMU sensors included 3-dimensional acceleration and gyroscope signals.

Thirty healthy participants (18 males, 12 females, aged 21.97 2.75 years; height 1.73 ± 0.10 m; weight 72.92 ± 12.57 kg) provided written informed consent (Auburn University IRB protocol no. 17-279-MR 1707 approved on 6 August 2023) and participated in the study at the Auburn University Biomechanical Engineering Laboratory. Seventy-nine 14 mm markers were placed on participants using the point cluster method (Figure 1) [29]. Ten motion-capture cameras positioned around the capture volume recorded marker trajectories with a sampling frequency of 120 Hz (Vicon, Oxford, UK). Eight IMU sensors (Delsys Trigno IM, Delsys Inc., Boston, MA, USA) to record 3-dimensional acceleration and angular velocity with a sampling frequency of 148 Hz were attached bilaterally to the participant’s feet, shank, thighs, and torso (Figure 1). Participants performed three repetitions of five actions in a motion-capture setting. The actions performed were backward walking, kneeling down, kneeling up, running, and walking.

**Figure 2:**
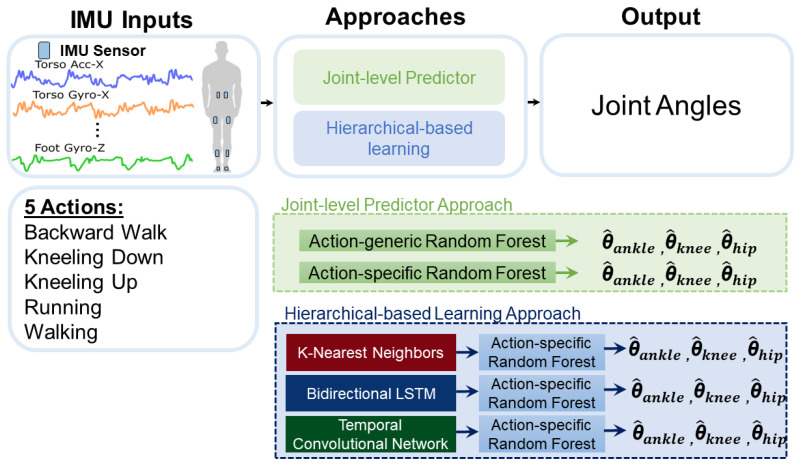
The proposed system utilized IMU sensor configurations for action classification and joint-level prediction. Eight IMU sensors were used for the action classifier stage, and two IMU inputs were used for the joint-level prediction stage. The selected IMU signals for the joint-specific random forest algorithm were analyzed based on sensors on the ipsilateral joint’s distal and proximal limb, with torso and thigh sensors used for hip angle prediction, thigh and shank for knee angle prediction, and shank and foot for ankle angle prediction.

The Grood and Suntay joint coordinate system was chosen to obtain joint angles, along with Visual3D software (v6 x64, C-Motion, Germantown, MD, USA) [30]. The IMU data were downsampled to 120 Hz to match the frequency of the motion capture. This enabled the machine learning models to train with one-to-one mapping between the inputs (IMU signals) and the targets (e.g., joint angles). Additionally, we synchronized the IMU sensors to the motion-capture system by aligning the motion-capture body segments to the IMU segments’ resultant velocity vectors. The overall workflow for the study is shown in Figure 2.

Eight IMU sensors (3-dimensional accelerometers and gyroscopes) were used as inputs to the models, yielding 48 input signals. The first stage of the hierarchical-based approach used these 48 signals for action classification. Although previous research suggested that the inclusion of joint angle inputs enhanced prediction accuracy for simple movements (e.g., ankle, knee, and hip flexion/extension) [12], preliminary analysis did not result in similar outcomes during more complex movements (e.g., backward walking, kneeling down, kneeling up, running, and walking). Consequently, we selected IMU inputs alone for the action-specific tier for this study. IMU inputs alone were also the most practical and flexible solution, since this configuration did not depend on multiple types of sensors. 

Several approaches to joint angle prediction were implemented in this study. The action-generic approach used a joint-level random forest algorithm trained on the five actions in this study to directly predict joint angles from IMU inputs (Figure 2). Three action-generic models were trained on joint-specific data to predict ankle, knee, or hip angles, respectively. These models used IMU sensors positioned both distal and proximal to the neighboring joint to predict future joint angles. Since each IMU sensor produced six signals (e.g., 3-dimensional accelerometry and angular velocity), the input features for the action-generic models were structured as M×N arrays, where M represents the number of time stamps in the sliding window of input feature data and N represents the number of input signals (12 in this case) across the five activities. Consequently, each action-generic model used 12 IMU input signals for predicting the joint angles. A trial-level split between training and testing data was performed for the joint-level models; two trials were used for training and one trial was used for testing. This was carried out for each combination of action, joint, and participant. The specific trials used for training and testing were randomly selected to avoid overfitting the models to particular trials during the process of data collection. Additionally, we trained an action-specific random forest classifier, assuming 100% accuracy of action classification (Figure 2). By assuming perfect classification accuracy, we were able to assess the optimal performance of the hierarchical approach. This approach was useful for evaluating the capabilities of both the action-level and joint-level algorithms, as it eliminated any confusion about whether improvements were due to the action-level model, the joint-level model, or a combination of both. The final approach used in this study was the hierarchical-based approach, which combined an action-level classifier with a joint-level predictor for tiered motion assessment. This approach is akin to ensemble modeling, where individual models are trained and stacked sequentially. In our study, models were trained for each action, and a meta-model (e.g., a classifier) determined the appropriate action-specific model for joint-level prediction. 

We employed three different classifiers of varying complexity for the hierarchical-based approach. The first and simplest classifier was the k-nearest neighbors (KNN) model, a non-parametric machine learning model that classifies data based on the majority class of its ‘k’ nearest neighbors, where ‘k’ is a pre-selected integer. The KNN method is often used for its excellent flexibility and interpretability. However, it can be sensitive to noise and outliers, and selecting the optimal k-value can be tedious and time-consuming. Additionally, the model is computationally expensive because it calculates the distance to every data point in the training set to determine the class of the nearest neighbors. The KNN model used in this study was based on the default values from the scikit-learn Python library, with the number of neighbors set to five. This choice was made to establish a simple baseline model as a starting point for developing the hierarchical approach. 

**Figure 3:**
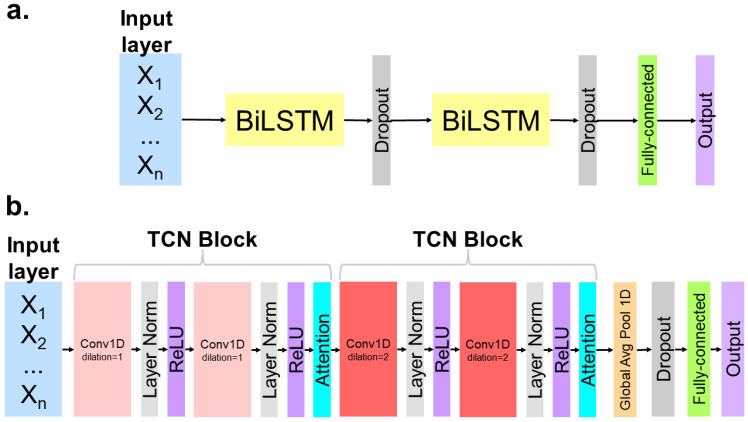
Model architecture for (**a**) BiLSTM and (**b**) TCN. The input layer comprised IMU signals from a sliding window. The output prediction was label encoded for the five activities used in this study (e.g., backward walking, kneeling down, kneeling up, running, and walking). The probability score from logits determined the resulting predicted action.

The second classifier we used for the hierarchical-based approach was a bidirectional long short-term memory (BiLSTM) model. LSTM-based models are commonly used for temporal-based prediction problems [31,32]. We utilized a bidirectional architecture since this was previously shown to be the most robust performer for predicting lower-limb joint angles during level-ground walking [24]. We followed a similar architecture to that employed by Hollinger et al. in 2023, using two BiLSTM layers, each with 100 neurons [24]. We also added dropout layers following each BiLSTM layer, with a dropout probability of 0.5 to prevent overfitting (Figure 3). Weight initialization was performed using He normal initialization for each BiLSTM layer to stabilize the gradients throughout the network during training. This technique initializes the weights using samples drawn from a normal distribution with zero mean and variance and leads to faster convergence in networks with many layers [33,34]. 

Lastly, we implemented a temporal convolutional network (TCN) classifier model with a similar structure that that used to estimate hip motion when traversing ramps or stairs and during level-ground locomotion (Figure 3) [9]. Each TCN block consisted of two 1-dimensional convolutional layers, each followed by a normalization layer and a rectified linear unit (ReLU) layer. The dilation rate increased with each block, represented by a darker gradient, which allowed the network’s receptive field to exponentially increase as data flowed through the network. An attention layer was used at the end of each TCN block to stabilize training. A global average pooling layer was used, followed by a dropout layer with a dropout rate of 0.5 and a fully connected dense layer at the end of the model. and was fed into the output layer to make the final action classification prediction.

The BiLSTM and TCN were trained for 200 epochs, which was similar to the TCN training performed by Molinaro et al. in 2022 [9]. A patience parameter of 50 epochs was applied to enable early stopping, which prevented overfitting to the training data by halting training when the validation loss failed to decrease for 50 consecutive epochs [9,35]. Each classifier (e.g., KNN, BiLSTM, and TCN) was trained, validated, and tested on IMU data from subject-wise splitting, where 20 subjects were used for training, five for validation, and five for testing. This was equal to approximately 66.7%, 16.7%, and 16.7% training, validation, and testing, to guarantee strong model performance and generalization. Hyperparameters, including the KNN algorithm’s number of neighbors and the neural network’s learning rate, were adjusted using the five subjects from the validation set. The best hyperparameters were found by assessing the model’s performance on this set during training. The testing set was excluded from training and was used to evaluate the model’s final performance on the held-out data. The subject-wise split ratios for the training, validation, and testing sets were chosen to prevent the model from overfitting to the training data. 

We implemented a sliding window approach, which is a commonly used method of dividing the sensor signals into smaller data segments so that they can be used as model input features. Each timestep for the joint-level predictors was represented by one motion-capture frame and was approximately 8 ms (i.e., 1/120 Hz). The sliding window comprised an input window (30 timesteps or ~250 ms) and an output prediction window (1 timestep or ~8 ms), with a sliding size of 1 timestep or ~8 ms and prediction horizon of 12 timesteps or 100 ms into the future. The same parameters were used for the classifiers, except for the sliding size, which was set to 87 IMU frames (e.g., ~40 ms) We followed a similar sliding window approach to that of Zaroug, 2022, but customized it to handle multiple input features (e.g., 48 signals from eight IMU sensors in our case) [36]. We evaluated the joint-level predictors using root mean squared error (RMSE) (Equation (1)).

(1) $$RMSE=\sqrt{\frac{\sum_{i=1}^{N}{{(\hat{\theta }}_{i}-\theta_{i})}^{2}\;}{N}}$$

where *N* is the number of data points, $\theta_{i}$ is the actual joint angle at the *i*th data point, and ${\hat{\theta }}_{i}$ is the predicted joint angle at the *i*th data point.

Classifier performance was evaluated by the number of true positives (*TP*), true negatives (*TN*), false positives (*FP*), and false negatives (*FN*) and was used to determine *precision*, *recall*, *F*1 score, and accuracy.

(2) $$Precision=100\times \frac{TP}{TP+FP}$$

(3) $$Recall=100\times \frac{TP}{TP+FN}$$

(4) $$F1\;Score=100\times \frac{2\times precision\times recall}{precision+recall}$$

(5) $$Accuracy=\frac{TP+TN}{TP+TN+FP+FN}$$

We also report *macro precision*, *macro recall*, and *macro F*1 *scores*, representing the average without considering the proportion for each action in the dataset (Equations (6)–(8)).

(6) $$Macro\;Precision=\frac{1}{n}\sum_{i=1}^{n}{Precision}_{i}$$

(7) $$Macro\;Recall=\frac{1}{n}\sum_{i=1}^{n}Recall$$

(8) $$Macro\;F1\;Score=\frac{1}{n}\sum_{i=1}^{n}{F1\;Score}_{i}$$

*Weighted precision*, *weighted recall*, and *weighted F*1 *scores* are reported and represent the averages weighted by the proportion of each action in the dataset (Equations (9)–(11)). These metrics accounted for class imbalance by emphasizing classes with more samples. The weighted metrics were employed to evaluate the models with actions that most closely resembled the class imbalance present in the dataset.

(9) $$Weighted\;Precision=\frac{\sum_{i=1}^{n}{Support}_{i}\times {Precision}_{i}}{\sum_{i=1}^{n}{Support}_{i}}$$

(10) $$Weighted\;Recall=\frac{\sum_{i=1}^{n}{Support}_{i}\times {Recall}_{i}}{\sum_{i=1}^{n}{Support}_{i}}$$

(11) $$Weighted\;F1\;Score=\frac{\sum_{i=1}^{n}{Support}_{i}\times {F1\;Score}_{i}}{\sum_{i=1}^{n}{Support}_{i}}$$

where n represents the total number of classes, ${Precision}_{i}$ represents the precision in class i, ${Recall}_{i}$ represents the recall in class i, ${F1\;Score}_{i}$ represents the *F*1 score in class i,  and ${Support}_{i}$ represents the number of true instances in class i.

A repeated-measures analysis of variance (ANOVA) was used to compare RMSE values between different models for each action. The mean RMSE across the 30 participants was analyzed for each relevant combination of models and conditions. The five conditions included in the ANOVA were action-specific random forest, action-generic random forest, KNN + action-specific random forest, BiLSTM + action-specific random forest, and TCN + action-specific random forest. These comparisons were repeated across five actions, with a significance threshold value set at 0.05. Post hoc pairwise Tukey honest significant difference (HSD) tests were performed following a significant ANOVA result for pairwise comparisons of the mean *RMSE* for each approach. Adjusted *p*-values (i.e., *p*-adj) were used during post hoc testing to avoid type I errors due to multiple pairwise comparisons. Partial eta squared (η^2^) values were calculated to estimate effect size, with thresholds 0.01, 0.06, and 0.14 representing small, medium, and large effects, respectively [37]. Greater η^2^ values corresponded to larger differences between measures of the central tendency of the action-generic, combined (KNN + random forest, BiLSTM + random forest, and TCN + random forest), and action-specific predictors. All statistical analyses were performed using Python’s stats module from the SciPy library, which includes a wide range of statistical functions. 

## 3. Results

**Figure 4:**
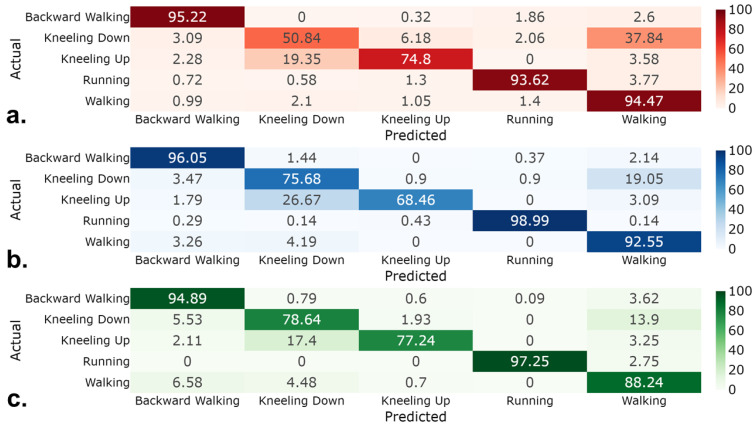
The classification performance from IMU inputs illustrated in confusion matrices for (**a**) KNN, (**b**) BiLSTM, and (**c**) TCN. The *y*-axis represents the true action, while the *x*-axis shows the predicted action. Darker shades along the diagonal indicate higher accuracy, while misclassifications appear off the diagonal. Units express percentages.

**Figure 5:**
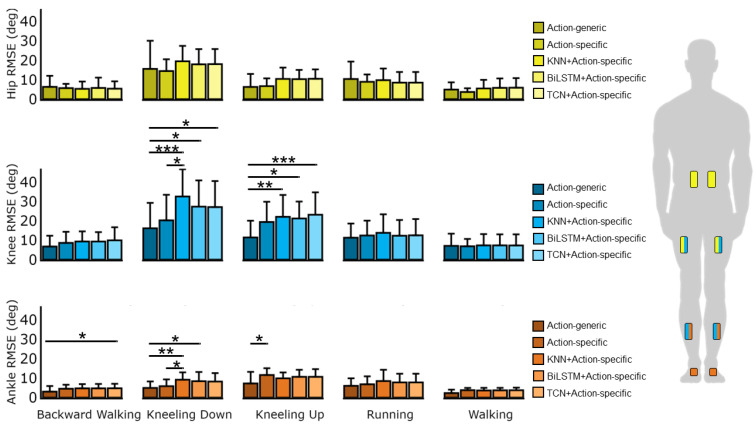
A comparison of prediction approaches across actions for the hip, knee, and ankle. In each subplot, the prediction approaches are shown in gradient colors from dark to light shades, representing action-generic, action-specific, KNN + action-specific, BiLSTM + action-specific, and TCN + action-specific. All joint-level prediction algorithms (e.g., action-generic and action-specific) utilized random forest to predict joint angles. The right panel shows the joint-specific IMU sensor configuration used to predict hip, knee, and ankle angles. The bar plots represent the mean prediction RMSE across all participants and the bars represent one standard deviation. The comparison bars group the prediction approaches based on post hoc pairwise Tukey HSD tests, showing significant results denoted as * *p* < 0.05, ** *p* < 0.01, and *** *p* < 0.001.

We evaluated the classification accuracy of the KNN, BiLSTM, and TCN classifiers for predicting the five actions. The BiLSTM and the TCN were trained for 53 and 57 epochs because the validation loss plateaued for 50 consecutive epochs. Figure 4 shows the confusion matrices of each classifier; the predicted and actual values indicate which actions were misclassified. Although the BiLSTM and TCN models had similar overall classification accuracy, the TCN performed better at distinguishing between similar actions, with greater accuracy for kneeling down (78.64% vs. 75.68%) and kneeling up (77.24% vs. 66.46%) (Figure 4). However, these differences did not result in significantly different RMSE for joint-angle prediction when paired with the action-specific random forest algorithm. For kneeling down, the comparative results were as follows: at the ankle, 8.45 ± 4.73 deg vs. 8.27 ± 4.35 deg; knee, 27.38 ± 13.54 vs. 27.15 ± 13.45 deg; hip, 17.96 ± 7.86 vs. 18.05 ± 7.79 deg. For kneeling up, the comparative results were as follows: at the ankle, 10.68 ± 3.62 vs. 10.70 ± 3.95 deg; knee, 21.27 ± 8.74 deg vs. 23.19 ± 11.61 deg; hip, 10.31 ± 4.76 vs. 10.50 ± 4.85 deg (Figure 5). Additionally, the misclassification rate of the KNN during kneeling down significantly worsened the RMSE for hierarchical-based prediction compared with the action-specific random forest algorithm (20.29 ± 13.22° vs. 32.61 ± 14.07° at the knee, *p* < 0.015) (Figure 5 and Table A5). The RMSE values (mean ± one standard deviation) displayed in Figure 5 are presented in tabular form in Appendix A (Table A1, Table A2 and Table A3).

The precision, recall, F1 scores, and accuracy of the classifiers are shown in Table 1, Table 2 and Table 3. The weighted average for each classifier exceeded the corresponding macro scores. This occurred because the highest misclassification rates were found in trials with the fewest predictions (e.g., kneeling down and kneeling up). Therefore, each classifier achieved higher weighted precision, recall, and F1 scores compared with their macro precision, recall, and F1 scores. ANOVA comparisons for action-generic, action-specific, and hierarchical-based approaches are presented in Table 4, Table 5 and Table 6. Significant ANOVA results prompted post hoc pairwise Tukey HSD tests, with the corresponding significant comparisons displayed as grouped comparison bars in Figure 5. The ANOVA results and post hoc pairwise Tukey HSD comparisons are provided in Table A1, Table A2, Table A3, Table A4, Table A5, Table A6, Table A7, Table A8 and Table A9 in Appendix A.

## 4. Discussion

The purpose of this study was to investigate the effectiveness of combining action-level and joint-level models for tiered motion assessment. We first hypothesized that an action-specific model would have greater accuracy than an action-generic model for joint-angle prediction. We tested this hypothesis by comparing the RMSE of joint-angle predictions 100 ms into the future, across five actions. Our results showed that the action-generic approach significantly outperformed the action-specific approach for predicting ankle angles during kneeling up (RMSE of 7.29 ± 5.96 deg vs. 11.63 ± 3.51 deg, *p* < 0.015) (Figure 5). Except for the kneeling-up trial, no other action resulted in a significant difference between the action-specific vs. action-generic prediction (Figure 5). Therefore, the first hypothesis that the action-specific model would have greater joint-angle prediction accuracy compared with the action-generic model was not supported by the results. 

Although statistically significant differences in prediction RMSE were observed, it is essential to further discuss the practical significance of these findings. For example, the reported effect sizes (i.e., partial η^2^) ranged from 0.009 to 0.152 across various actions and joints (Table 4, Table 5 and Table 6). Additionally, effect sizes of 0.139 and 0.152 were reported for the RMSE values for ankle and knee joints during kneeling down, which indicates a moderate to large effect of the model type on prediction accuracy. These findings suggest that changing the prediction approach (e.g., action-generic, action-specific, or hierarchical-based) can result in a meaningful difference in RMSE during challenging non-cyclic actions (e.g., kneeling down) [37]. However, the practical implications of these effect sizes in real-world applications in relation to user comfort and control of wearable assistive devices remain unclear and should be considered in future work. 

There are several reasons why our first hypothesis may have been rejected. First, the action-generic models were trained on more data than the action-specific models. The action-generic models were trained across multiple actions and participants, resulting in 55,683 training samples. This amount of data was approximately 130 times greater than the number of training samples used for the action-specific model, which came from subject-dependent tasks. Therefore, the additional training data from diverse sources (multiple actions and participants) could have enabled the action-generic model to learn more nuanced patterns of human motion and better capture the temporal relationship between IMUs and future joint angles. In contrast, the action-specific models were inherently limited due to using smaller amounts of less diverse training data. Although subject-independent data could increase the amount of training data by pooling samples across participants, this approach could potentially degrade performance due to variations in motion patterns between individuals, especially during more complex movements [38]. Therefore, the action-specific models were restricted to the specific actions on which they were trained. This constraint limited their ability to generalize, compared with action-generic models. Future work should address this inherent constraint by exploring how increased and more diverse training data could enhance the performance of action-specific models. Although the training of action-specific models using subject-dependent data followed a similar training paradigm to prior work that evaluated the effect of sensor combinations across simple movements, the limited training data may have prevented the models from learning the feature representations for joint-angle prediction during complex tasks, leading to underfitting [12]. 

We also hypothesized that a combination of action-level and joint-level prediction models (i.e., a hierarchical-based approach) would result in a more accurate prediction of joint angles compared with a joint-level-only prediction model. Given that the action-specific random forest method assumed perfect action classification, it should not be surprising that the hierarchical-based approach with imperfect action classifiers resulted in greater levels of error in prediction (Figure 5). The most obvious misclassifications occurred during the kneeling-down and kneeling-up trials, where KNN predicted with 50.84% and 74.80% accuracy, BiLSTM 75.68% and 68.46%, and TCN 78.64% and 77.24% (Figure 4). Although kneeling down and kneeling up contributed to the increased misclassification rates, the length of these trials was relatively shorter compared with the cyclic actions (e.g., backward walking, running, and walking), which resulted in the macro average being less than the weighted average for precision (84.66% vs. 86.29% for KNN, 89.58% vs. 90.40% for BiLSTM, and 89.32% vs. 89.44% for TCN), recall (81.16% vs. 86.59% for KNN, 86.34% vs. 89.87% for BiLSTM, and 87.25% vs. 89.30% for TCN), and F1 score (82.26% vs. 85.94% for KNN, 87.40% vs. 89.83% for BiLSTM, and 88.14% vs. 89.29% for TCN) (Table 1, Table 2 and Table 3). 

Although the TCN’s ability to distinguish between kneeling down and kneeling up was better than that of the BiLSTM, it did not result in a significantly different RMSE for joint-angle prediction when fed into the subsequent action-specific random forest algorithm. Thus, that neither classifier significantly reduced prediction RMSE when combined with action-specific joint-level models. However, the misclassification rate of the KNN during kneeling down significantly worsened the RMSE for hierarchical-based prediction compared with the action-specific random forest (20.29 ± 13.22° vs. 32.61 ± 14.07° at the knee, *p* < 0.015); a misclassification rate of 25.2% during kneeling down increased prediction RMSE in relation to the ankle and knee joins by a mean 3.32° and 12.32°, which was statistically significant (*p* < 0.048, *p* < 0.015) (Figure 4 and Figure 5). As shown in Figure 4, kneeling down was misclassified as walking in 37.84%, 19.04%, and 13.90% of cases by the KNN, BiLSTM, and TCN models, respectively. Additionally, these models misclassified kneeling up as kneeling down at rates of 19.35%, 26.67%, and 17.40%. These misclassification reflect how the models struggled to correctly classify non-cyclic actions (e.g., kneeling up and kneeling down) compared with cyclic actions (e.g., backward walking, running, walking) (Figure 4).

There are several reasons why increased misclassification rates occurred during certain tasks such as kneeling down and kneeling up. For instance, the differences in model architecture could have played a role in certain classifiers’ ability to effectively distinguish between similar features of adjacent actions. For example, the KNN’s lower classification rates (e.g., 50.84% and 74.80% for kneeling down and kneeling up) stemmed from its sensitivity to overlapping feature spaces, as seen in Figure 4. Although different model architectures leveraging sequential layers demonstrated improved accuracy for kneeling down and kneeling up (e.g., 75.68% and 68.46% for BiLSTM, and 78.64% and 77.24% for TCN), their errors persisted due to the challenges inherent in predicting non-cyclic motion patterns. As shown in Table 1, Table 2 and Table 3, the classification metrics for kneeling down and kneeling up were lower compared with those for cyclic actions. This can be partially attributed to the presence of fewer samples of non-cyclic actions in the training dataset, resulting in reduced representation during model training. Addressing this imbalance by collecting additional non-cyclic task data could improve classification performance. Since the movement patterns of certain non-cyclic actions overlapped with other actions, they resulted in higher misclassification rates. For example, the early phase of kneeling down closely resembles walking; this caused the classifiers to confuse these actions (Figure 4). To address these limitations in future studies, we recommend exploring strategies such as increasing the diversity and quantity of training samples for non-cyclic actions and implementing transition-aware classification models to better handle ambiguous phases of motion. 

The broader implications of this work highlight the potential danger of using a simple KNN classifier, where misclassified actions significantly worsen subsequent joint-level predictions at the ankle and hip during kneeling-down tasks. Moreover, even more complex classifiers such as the BiLSTM and TCN did not provide significantly improved results when combined with the joint-level model. As a result, neither the BiLSTM nor the TCN classifier significantly differed from a perfect action-specific classifier. This analysis shows how even with perfect action classification accuracy from the BiLSTM or TCN, joint-angle prediction RMSE would still not have significantly improved. Under the specific conditions and actions used in this study, the action-generic random forest model was the best approach for predicting future joint angles in 10 out of 60 compared scenarios (e.g., five actions × four prediction approaches) (Figure 5). However, this may not always be the case in different contexts or datasets involving other actions and sensors.

Accuracy and loss during training showed similar patterns for the BiLSTM and the TCN. The BiLSTM trained for 53 epochs, while the TCN trained for 57 epochs. Since the patience parameter for early stopping was set to 50 epochs, the validation accuracy did not improve after three epochs for the BiLSTM and seven epochs for the TCN. The patience parameter of 50 was similar to that used by Molinaro et al. in 2022; however, their TCN model did not use an attention layer and was trained on locomotor activity (e.g., level ground, ramp incline/decline, and stairs ascent/descent) [9]. Reducing the patience parameter could help mitigate the risk of overfitting to the training data and should be explored in future studies.

This study had several limitations. First, we included only five actions, while activities of daily living encompass a wider variety of actions beyond those used in this study. Although including only five actions is a limitation of this work, our approach shows promise for effectively training a task-agnostic joint-level predictor. The improved RMSE for joint-angle prediction compared with an action-specific approach suggests that the inclusion of additional actions could improve multi-action intent prediction even further. Future work should explore expanding the set of actions included in this study. The misclassification rates during kneeling down and kneeling up may have been the result of predicting non-cyclic actions compared with the cyclic actions of walking, running, and backward walking. Incorporating a wider range of non-cyclic tasks from additional datasets could potentially improve the accuracy of action classification with non-cyclic motion [39]. Including additional actions alongside a combination of subject-dependent and subject-independent factors may also be worth exploring. Open-source biomechanical datasets with diverse actions could make this possible [39,40]. Another limitation of this work was the similarity among actions. For instance, the initial phase of kneeling down is similar to walking; the discrepancy from walking begins to occur as the participant begins to kneel in a downward vertical motion. Future studies should consider incorporating a transition period where classification is not so severely penalized compared with non-transition periods. Another important limitation of this study is that the data were collected from healthy young participants in a controlled laboratory setting and analyzed offline. Expanding the dataset to include participants from a wider age range could improve the model’s applicability for age-specific scenarios (e.g., fall detection among the elderly). Additionally, further testing in real-time environments is necessary, since practical issues such as prediction delays, sensor noise, and computational overhead could influence the practical implementation of these models in wearable devices. For example, BiLSTM and TCN models introduce greater computational costs due to their reliance on sequential data processing and multiple layers in their architecture [41]. Conversely, random forest models leverage simpler ensemble-based methods which operate faster with less computational demand. In practice, simpler models like random forest or KNN are suitable for real-time systems where computational efficiency is critical. Advanced models (e.g., BiLSTM and TCN) offer a high level of accuracy but may require additional resources, such as GPU accelerators, to satisfy real-time constraints of wearable devices. These differences in model complexity could affect latency and computational overhead when deployed in a real-time system. Prior work involving a comparative analysis of runtimes between kinematic and random forest models reported how random forest models introduced longer runtimes but were more accurate compared with kinematic models for predicting joint angles [16]. Future work performing similar analysis on more advanced models would help identify the trade-offs between accuracy and resource consumption for controlling wearable exoskeletons in real-time settings. 

Future work building upon the current research should focus on expanding the dataset to include a broader range of actions (e.g., non-cyclic tasks) to improve classification accuracy. Additionally, exploring a balanced dataset with equal samples for action-specific and action-generic models with increased repetition of actions could provide a fairer comparison of their performances. Since this offline analysis was limited to a lab-based environment, future research should prioritize online testing to validate these models in real-world contexts. Expanding the participant pool to include diverse age groups and conducting studies in less-controlled environments could further improve the model’s generalizability for various assistive technologies. Although the action-generic random forests showed the best results across the board in this study, future work should consider alternative joint-level models with greater complexity, such as LSTM or TCN. These complex models are typically used for sequence-to-sequence problems and could potentially capture the IMU feature representations of joint angles more effectively. From this work, we observed that misclassification worsened the prediction accuracy (Figure 5). However, not all misclassified actions worsened the prediction accuracy by equal amounts. When the classifier misclassified actions similar to the true action, the joint-level prediction did not worsen so much compared with more dissimilar actions. Therefore, future studies should consider tuning algorithms to predict actions grouped by their similarities as quantified in distinct clusters. Recognizing similarities between actions could also benefit assistive technology for smoother transitions between certain actions (e.g., walk to jog, jog to walk, walk to turn).

The results of this study highlight how large open-source datasets could be critical for enabling versatile, scalable, and robust control of wearable assistive devices, as they allow machine learning models to leverage task-agnostic control and facilitate between-device transfer learning [39]. Multi-action intent recognition is complex and often deals with the challenge of predicting nonlinear targets. Although machine learning is well suited for solving nonlinear problems with large amounts of training data, certain models perform better than others at predicting certain actions and joint movements. Therefore, a one-size-fits-all approach should be avoided when developing solutions for multi-action intent recognition. 

## 5. Conclusions

This study explored whether a combination of action-level and joint-level models would improve the accuracy of joint-angle prediction compared with a single action-generic joint-level model. Although the action-level KNN, BiLSTM, and TCN approaches predicted the five actions with overall accuracy of 86.59%, 89.87%, and 89.30%, respectively, they did not significantly enhance the accuracy of joint-angle prediction compared with the action-generic model. Furthermore, even the action-specific predictor, which assumed perfect classification accuracy, did not outperform the action-generic model. These findings suggest that accurate prediction of joint angles depends more on the amount and diversity of data than on action-specific data. The amount of training data fed into the action-generic model was approximately 130 times larger than the action-specific and the hierarchical-based models, because the model was task-agnostic and subject-independent. The results of this study suggest that incorporating disparate data sources can improve the prediction capability of machine learning models, even compared with more relevant but smaller datasets [42]. Joint-angle prediction across multiple actions is complex, and models may require a large amount of diverse training data to capture the wide range of motion patterns. The action-generic model in this study leveraged increased data quantity and diverse features to capture the nuanced patterns of human motion, resulting in improved joint-angle predictions overall.

## Figures and Tables

**Table 1:** Classification results for KNN. Units represent percentages.

KNN	Precision	Recall	F1 Score
Backward Walking	95.09	95.40	95.25
Kneeling Down	72.18	49.42	58.67
Kneeling Up	86.21	73.17	79.16
Running	89.44	93.33	91.35
Walking	80.38	94.47	86.85
Accuracy			86.59
Macro avg	84.66	81.16	82.26
Weighted avg	86.29	86.59	85.94

**Table 2:** Classification results for BiLSTM. Units represent percentages.

BiLSTM	Precision	Recall	F1 Score
Backward Walking	95.57	96.05	95.81
Kneeling Down	58.69	75.68	72.01
Kneeling Up	97.68	68.46	80.50
Running	97.85	98.99	98.41
Walking	88.13	92.55	90.28
Accuracy			89.87
Macro avg	89.58	86.34	87.40
Weighted avg	90.40	89.87	89.83

**Table 3:** Classification results for TCN. Units represent percentages.

TCN	Precision	Recall	F1-Score
Backward Walking	92.36	94.89	93.61
Kneeling Down	75.25	78.64	76.90
Kneeling Up	92.23	77.24	84.07
Running	99.70	97.25	98.46
Walking	87.07	88.24	87.65
Accuracy			89.30
Macro avg	89.32	87.25	88.14
Weighted avg	89.44	89.30	89.29

**Table 4:** ANOVA table comparing hip angle prediction RMSE for action-generic, action-specific, and hierarchical-based approaches. Emboldened *p*-value denotes a statistically significant difference.

Action	Joint	Sum sq.	df	F-Stat	*p*-Value	Partial Eta sq.
Backward Walking	Hip	22.375	4	0.323	0.862	0.009
Kneeling Down	Hip	267.710	4	0.763	0.551	0.025
Kneeling Up	Hip	311.398	4	2.645	**0.037**	0.087
Running	Hip	147.175	4	1.383	0.243	0.037
Walking	Hip	85.65	4	1.261	0.288	0.035

**Table 5:** ANOVA table comparing knee angle prediction RMSE for action-generic, action-specific, and hierarchical-based approaches. Emboldened *p*-values denote statistically significant differences.

Action	Joint	Sum sq.	df	F-Stat	*p*-Value	Partial Eta sq.
Backward Walking	Knee	168.826	4	1.267	0.286	0.036
Kneeling Down	Knee	4225.085	4	5.572	**<0.001**	0.152
Kneeling Up	Knee	2133.237	4	4.889	**0.001**	0.142
Running	Knee	262.43	4	1.007	0.406	0.028
Walking	Knee	82.613	4	0.801	0.526	0.023

**Table 6:** ANOVA table comparing ankle angle prediction RMSE for action-generic, action-specific, and hierarchical-based approaches. Emboldened *p*-values denote statistically significant differences.

Action	Joint	Sum sq.	df	F-Stat	*p*-Value	Partial Eta sq.
Backward Walking	Ankle	63.393	4	2.794	**0.028**	0.072
Kneeling Down	Ankle	306.994	4	4.608	**0.002**	0.139
Kneeling Up	Ankle	223.130	4	3.212	**0.0155**	0.105
Running	Ankle	82.216	4	0.963	0.430	0.026
Walking	Ankle	8.886	4	0.958	0.433	0.026

## Data Availability

The raw data supporting the conclusions of this article will be made available by the authors on request.

## Appendix A

The appendix includes additional tables showing values from Figure 5 (Table A1, Table A2 and Table A3) along with statistical analyses performed in this study (Table A4, Table A5, Table A6, Table A7, Table A8 and Table A9). Post hoc pairwise Tukey HSD test results are shown for ANOVA comparisons that were statistically significant.

**Table A1:** Comparison of ankle prediction RMSE (mean ± one standard deviation) for various conditions.

Hip RMSE	Action-Generic	Action-Specific	KNN + Action-Specific	BiLSTM + Action-Specific	TCN + Action-Specific
Backward Walking	6.33 ± 5.69	4.69 ± 2.20	5.31 ± 3.77	5.78 ± 5.33	5.38 ± 3.82
Kneeling Down	15.60 ± 14.49	15.72 ± 6.06	19.50 ± 7.95	17.96 ± 7.86	18.05 ± 7.79
Kneeling Up	6.28 ± 6.71	7.99 ± 4.03	10.44 ± 5.85	10.31 ± 4.76	10.50 ± 4.85
Running	10.40 ± 8.95	7.19 ± 3.77	9.81 ± 5.99	8.53 ± 5.46	8.53 ± 5.46
Walking	4.96 ± 3.70	4.03 ± 1.92	5.50 ± 4.44	5.86 ± 4.83	5.92 ± 4.91

**Table A2:** Comparison of knee prediction RMSE (mean ± one standard deviation) for various conditions.

Knee RMSE	Action-Generic	Action-Specific	KNN + Action-Specific	BiLSTM + Action-Specific	TCN + Action-Specific
Backward Walking	6.79 ± 5.57	8.63 ± 5.74	9.37 ± 5.22	9.34 ± 4.88	9.97 ± 6.75
Kneeling Down	16.22 ± 13.14	20.29 ± 13.22	32.61 ± 14.07	27.38 ± 13.54	27.15 ± 13.45
Kneeling Up	11.49 ± 8.63	19.43 ± 10.48	22.14 ± 11.25	21.27 ± 8.74	23.19 ± 11.61
Running	11.33 ± 7.34	12.48 ± 7.67	13.84 ± 9.59	12.38 ± 8.11	12.54 ± 8.42
Walking	7.10 ± 6.30	6.90 ± 3.80	7.37 ± 5.81	7.36 ± 5.73	7.35 ± 5.72

**Table A3:** Comparison of ankle prediction RMSE (mean ± one standard deviation) for various conditions.

Ankle RMSE	Action-Generic	Action-Specific	KNN + Action-Specific	BiLSTM + Action-Specific	TCN + Action-Specific
Backward Walking	3.11 ± 2.86	4.60 ± 2.05	4.78 ± 2.19	4.78 ± 2.25	4.83 ± 2.29
Kneeling Down	4.99 ± 3.32	5.90 ± 3.49	9.22 ± 3.77	8.45 ± 4.73	8.27 ± 4.35
Kneeling Up	7.29 ± 5.96	11.63 ± 3.51	9.91 ± 3.07	10.68 ± 3.62	10.70 ± 3.95
Running	6.06 ± 3.89	6.86 ± 4.10	8.55 ± 5.75	7.81 ± 4.50	7.81 ± 4.50
Walking	2.36 ± 1.76	3.83 ± 1.18	3.71 ± 1.31	3.76 ± 1.29	3.82 ± 1.34

**Table A4:** Pairwise Tukey HSD comparing various conditions of ankle prediction RMSE for backward walking. The table presents the mean difference (deg), adjusted *p*-value (*p*-adj), and the 95% confidence interval as upper and lower bounds. Emboldened *p*-adj value denotes a statistically significant difference.

Group 1	Group 2	MEAN DIFF	*p*-adj	Lower	Upper
Action-generic	Action-specific	1.487	0.1274	−0.241	3.2148
Action-generic	BiLSTM + action-specific	1.663	0.056	−0.024	3.349
Action-generic	KNN + action-specific	1.665	0.065	−0.063	3.392
Action-generic	TCN + action-specific	1.716	**0.047**	0.016	3.416
Action-specific	BiLSTM + action-specific	0.176	0.999	−1.511	1.862
Action-specific	KNN + action-specific	0.178	0.999	−1.550	1.905
Action-specific	TCN + action-specific	0.229	0.999	−1.471	1.929
BiLSTM + action-specific	KNN + action-specific	0.002	1.000	−1.685	1.689
BiLSTM + action-specific	TCN + action-specific	0.053	1.000	−1.605	1.711
KNN + action-specific	TCN + action-specific	0.051	1.000	−1.648	1.751

**Table A5:** Pairwise Tukey HSD comparing various conditions of knee prediction RMSE for kneeling down. The table presents the mean difference (deg), adjusted *p*-value (*p*-adj), and the 95% confidence interval as upper and lower bounds. Emboldened *p*-adj values denote statistically significant differences.

Group 1	Group 2	Mean Diff	*p*-adj	Lower	Upper
Action-generic	Action-specific	4.077	0.843	−6.926	15.079
Action-generic	BiLSTM + action-specific	11.167	**0.036**	0.474	21.859
Action-generic	KNN + action-specific	16.394	**<0.001**	5.701	27.087
Action-generic	TCN + action-specific	10.933	**0.042**	0.241	21.626
Action-specific	BiLSTM + action-specific	7.090	0.358	−3.603	17.783
Action-specific	KNN + action-specific	12.317	**0.015**	1.625	23.010
Action-specific	TCN + action-specific	6.857	0.393	−3.836	17.549
BiLSTM + action-specific	KNN + action-specific	5.227	0.632	−5.146	15.601
BiLSTM + action-specific	TCN + action-specific	−0.233	1.000	−10.607	10.140
KNN + action-specific	TCN + action-specific	−5.461	0.592	−15.834	4.913

**Table A6:** Pairwise Tukey HSD comparing various conditions of ankle prediction RMSE for kneeling down. The table presents the mean difference (deg), adjusted *p*-value (*p*-adj), and the 95% confidence interval as upper and lower bounds. Emboldened *p*-adj values denote statistically significant differences.

Group 1	Group 2	Mean Diff	*p*-adj	Lower	Upper
Action-generic	Action-specific	0.910	0.947	−2.501	4.320
Action-generic	BiLSTM + action-specific	3.468	**0.035**	0.161	6.774
Action-generic	KNN + action-specific	4.233	**0.005**	0.926	7.540
Action-generic	TCN + action-specific	3.285	0.052	−0.022	6.592
Action-specific	BiLSTM + action-specific	2.558	0.209	−0.749	5.865
Action-specific	KNN + action-specific	3.324	**0.048**	0.017	6.631
Action-specific	TCN + action-specific	2.375	0.277	−0.931	5.682
BiLSTM + action-specific	KNN + action-specific	0.766	0.964	−2.434	3.965
BiLSTM + action-specific	TCN + action-specific	−0.183	0.999	−3.382	3.017
KNN + action-specific	TCN + action-specific	−0.948	0.924	−4.148	2.251

**Table A7:** Pairwise Tukey HSD comparing various conditions of hip prediction RMSE for kneeling up. The table presents the mean difference (deg), adjusted *p*-value (*p*-adj), and the 95% confidence interval as upper and lower bounds.

Group 1	Group 2	Mean Diff	*p*-adj	Lower	Upper
Action-generic	Action-specific	1.709	0.857	−3.049	6.467
Action-generic	BiLSTM + action-specific	4.037	0.103	−0.477	8.551
Action-generic	KNN + action-specific	4.160	0.086	−0.354	8.673
Action-generic	TCN + action-specific	4.226	0.078	−0.288	8.740
Action-specific	BiLSTM + action-specific	2.328	0.610	−2.186	6.842
Action-specific	KNN + action-specific	2.451	0.561	−2.063	6.964
Action-specific	TCN + action-specific	2.517	0.535	−1.997	7.030
BiLSTM + action-specific	KNN + action-specific	0.123	1.000	−4.133	4.378
BiLSTM + action-specific	TCN + action-specific	0.189	0.999	−4.067	4.444
KNN + action-specific	TCN + action-specific	0.066	1.000	−4.190	4.322

**Table A8:** Pairwise Tukey HSD comparing various conditions of knee prediction RMSE for kneeling up. The table presents the mean difference (deg), adjusted *p*-value (*p*-adj), and the 95% confidence interval as upper and lower bounds. Emboldened *p*-adj values denote statistically significant differences.

Group 1	Group 2	Mean Diff	*p*-adj	Lower	Upper
Action-generic	Action-specific	7.948	0.070	−0.405	16.300
Action-generic	BiLSTM + action-specific	9.787	**0.012**	1.519	18.056
Action-generic	KNN + action-specific	10.653	**0.005**	2.385	18.922
Action-generic	TCN + action-specific	11.700	**<0.001**	3.431	19.968
Action-specific	BiLSTM + action-specific	1.840	0.972	−6.429	10.108
Action-specific	KNN + action-specific	2.706	0.894	−5.563	10.974
Action-specific	TCN + action-specific	3.752	0.718	−4.517	12.020
BiLSTM + action-specific	KNN + action-specific	0.866	0.998	−7.318	9.049
BiLSTM + action-specific	TCN + action-specific	1.912	0.967	−6.272	10.096
KNN + action-specific	TCN + action-specific	1.046	0.997	−7.137	9.230

**Table A9:** Pairwise Tukey HSD comparing various conditions of ankle prediction RMSE for kneeling up. The table presents the mean difference (deg), adjusted *p*-value (*p*-adj), and the 95% confidence interval as upper and lower bounds. Emboldened *p*-adj value denotes a statistically significant difference.

Group 1	Group 2	Mean Diff	*p*-adj	Lower	Upper
Action-generic	Action-specific	4.340	**0.011**	0.685	7.995
Action-generic	BiLSTM + action-specific	3.393	0.058	−0.074	6.861
Action-generic	KNN + action-specific	2.626	0.227	−0.842	6.093
Action-generic	TCN + action-specific	3.411	0.056	−0.057	6.878
Action-specific	BiLSTM + action-specific	−0.947	0.942	−4.414	2.521
Action-specific	KNN + action-specific	−1.715	0.647	−5.182	1.753
Action-specific	TCN + action-specific	0.929	0.946	−4.397	2.538
BiLSTM + action-specific	KNN + action-specific	−0.768	0.966	−4.037	2.501
BiLSTM + action-specific	TCN + action-specific	0.018	1.000	−3.252	3.287
KNN + action-specific	TCN + action-specific	0.785	0.963	−2.484	4.055
